# Nephron specific ATP6AP2 knockout increases urinary excretion of fatty acids and decreases renal cortical megalin expression

**DOI:** 10.1038/s41598-024-69749-x

**Published:** 2024-08-12

**Authors:** Silas A. Culver, Stefan R. Hargett, Jamie L. L. Q. Balugo, John J. Gildea, Thurl E. Harris, Helmy M. Siragy

**Affiliations:** 1 Division of Endocrinology and Metabolism, Department of Medicine, University of Virginia Health System, P.O. Box 801409, Charlottesville, VA 22908-1409 USA; 2 Department of Pharmacology, University of Virginia Health System, Charlottesville, USA; 3 Department of Pathology, University of Virginia Health System, Charlottesville, USA

**Keywords:** ATP6AP2, (pro)renin receptor, Lipid, Kidney, Obesity, Chronic kidney disease, Obesity, Metabolic syndrome, Dyslipidaemias

## Abstract

ATP6AP2 knockout in the renal nephron impairs receptor-mediated endocytosis, increasing urinary albumin and glucose excretion and impairing weight gain. Nonesterified fatty acids (NEFA) in urine are bound to albumin and reabsorbed in the proximal tubule through receptor-mediated endocytosis by the megalin–cubilin complex. We hypothesized that ATP6AP2 knockout increases urinary NEFA excretion through a reduction in megalin. Ten-week-old male C57BL/6 mice with nephron specific inducible ATP6AP2 knockout and noninduced controls were fed either normal diet (ND 12% fat) or high fat diet (HFD 45% fat) for 6 months. ATP6AP2 knockout significantly increased urine albumin:creatinine ratio in both ND and HFD fed mice while normalized urine NEFA concentration increased 489% and 259% in ND and HFD knockout mice compared to respective controls. Knockout decreased renal cortical megalin mRNA by 47% on ND and 49% on HFD while megalin protein expression decreased by 36% and 44% respectively. At the same time, markers of mTOR activity were increased while autophagy was impaired. Our results indicate that nephron specific ATP6AP2 knockout increases urinary NEFA excretion in the setting of impaired receptor-mediated endocytosis. Further investigation should determine whether ATP6AP2 contributes to obesity related ectopic lipid deposition in the proximal tubule.

## Introduction

Excess body weight has become a highly prevalent healthcare concern with more than 40% of the adult US population currently obese and another 30% overweight^1^. One important sequelae of obesity is an increased risk of impaired kidney function^2,3^. While the etiology of kidney injury in obesity is multifactorial, the role of excess lipid has gained recent attention as a risk factor for chronic kidney disease^4–6^. However, the mechanisms of lipid uptake by the kidney are incompletely understood.

Nonesterified fatty acids (NEFA) travel in the circulation bound to albumin. Albumin bound NEFA are filtered through the glomerulus and are subsequently taken up along with albumin by the renal proximal tubule epithelial cells via the megalin–cubilin complex in a process known as receptor-mediated endocytosis^7,8^. Inhibition of megalin–cubilin results in impaired proximal tubule uptake of albumin and increased albuminuria^9^.

ATP6AP2, previously known as the (pro)renin receptor, is expressed in all segments of the renal tubule including the proximal tubule epithelium^10,11^. In addition to its 39-kDa full length and 28-kDA soluble forms, ATP6AP2 is cleaved to form an 8.9-kDA accessory subunit of the vacuolar H^+^-ATPase that is necessary for acidification of intracellular vesicles and receptor-mediated endocytosis^12,13^. Our laboratory recently demonstrated that mice with renal proximal tubule ATP6AP2 knockout fail to develop obesity on high fat diet (HFD) and develop increased urinary excretion of albumin and glucose^10^. Others have similarly shown that loss of ATP6AP2 in the kidney causes albuminuria by impairing receptor-mediated endocytosis while V-ATPase inhibition in the drosophila wing reduces megalin expression^14,15^. However, whether albuminuria with ATP6AP2 knockout is also accompanied by greater urinary NEFA excretion is not known. In this study, we hypothesized that nephron specific ATP6AP2 knockout would result in increased urine NEFA and decreased renal cortical megalin expression.

## Results

### Renal cortical ATP6AP2 expression is reduced in the nephron specific ATP6AP2 knockout mouse

Our group and others have previously demonstrated that the Pax8-rtTA/LC-1 system utilized in this study is effective in reducing ATP6AP2 expression in both the proximal and distal nephron^10,16^. Consistent with our prior studies, RT-PCR demonstrated that ATP6AP2 knockout significantly decreased ATP6AP2 gene expression under both ND and HFD conditions by 46% and 38% respectively (p < 0.05) (Supplementary Fig. S1) There was, however, no significant difference in ATP6AP2 expression between ND and HFD controls.

### Nephron specific ATP6AP2 knockout prevented weight gain and increased urine NEFA excretion

**Figure 1:**
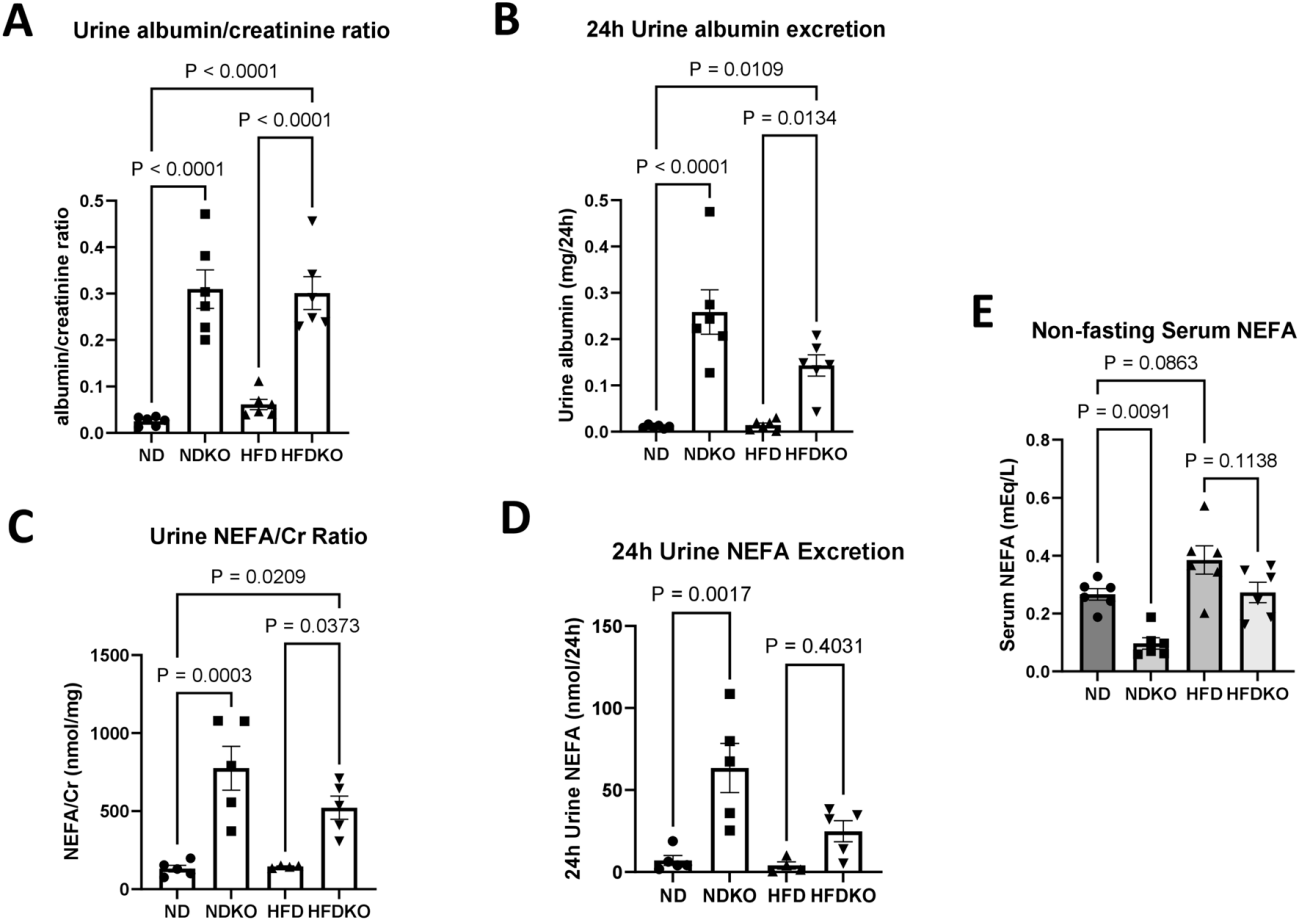
Urine albumin and NEFA excretion. Obtained after 6 months of ND vs HFD in mice with and without ATP6AP2 knockout. (**A**) Urine albumin to creatinine ratio. N = 6 for all groups, (**B**) 24-h urine albumin excretion N = 6 for all groups, (**C**) urine NEFA concentration normalized to urine creatinine. N = 5 for all groups, (**D**) 24-h urine NEFA excretion. N = 5 for all groups. (**E**) Serum NEFA levels under random fed conditions. N = 6 for all groups. Data presented as mean ± SEM.

We have previously shown that nephron specific ATP6AP2 knockout prevents HFD induced weight gain^10^. Mice in this study similarly demonstrated a 54% greater body weight with HFD alone compared to ND controls (p < 0.001) while NDKO mice had 17% lower body weight than ND controls (p < 0.01) and HFDKO mice weighed 43% less than control mice on HFD (p < 0.001) (Supplementary Fig. S2A). These differences in body weight corresponded to differences in insulin resistance (Supplementary Fig. S3). The nephron specific ATP6AP2 knockout mouse is also known to have polyuria due to nephrogenic diabetes insipidus^16^ and the knockout mice in this study also had increased 24-h urine volume on both ND and HFD compared to their respective controls (p < 0.05) (Supplementary Fig. S2B). We have also found that nephron specific knockout of ATP6AP2 produces albuminuria^10^. In the current study, there was no significant difference in albumin:creatinine ratio between mice on ND versus HFD as shown in Fig. 1A while ATP6AP2 KO resulted in a 12-fold increase in albumin:creatinine ratio in mice on ND (p < 0.001) and a fivefold increase in mice on HFD (p < 0.001). Similar to the previous study, 24 h urine albumin excretion was increased 23-fold in NDKO mice compared to ND controls (p < 0.001) and tenfold in HFDKO mice compared to HFD controls (Fig. 1B). NEFA are filtered through the glomerulus bound to albumin and contribute to fat deposition in the kidney in the setting of obesity^4^. Given the increased albuminuria in ATP6AP2 knockout mice, we investigated urine NEFA content to determine whether knockout mice also had increased urine NEFA excretion. As depicted in Fig. 1C, urine NEFA concentration normalized to urine creatinine increased by 489% in NDKO mice compared to ND controls (p < 0.01) while HFDKO mice had a 259% increase when compared to HFD controls (p < 0.05). Total 24-h urine NEFA excretion increased by 800% in NDKO over ND control mice (p < 0.01) (Fig. 1D) while HFDKO mice on average had a 520% increase in 24-h urine NEFA excretion though this did not reach statistical significance (p = 0.4). There was a nonsignificant 44% increase in serum NEFA in HFD fed control mice compared to ND controls (p = 0.08) (Fig. 1E). ATP6AP2 knockout reduced circulating NEFA levels by 63% in NDKO mice compared to ND controls (p < 0.01) while there was a nonsignificant decrease of 30% in HFDKO mice compared to HFD controls (p = 0.11).

### Nephron specific ATP6AP2 knockout reduced renal cortical megalin expression

**Figure 2:**
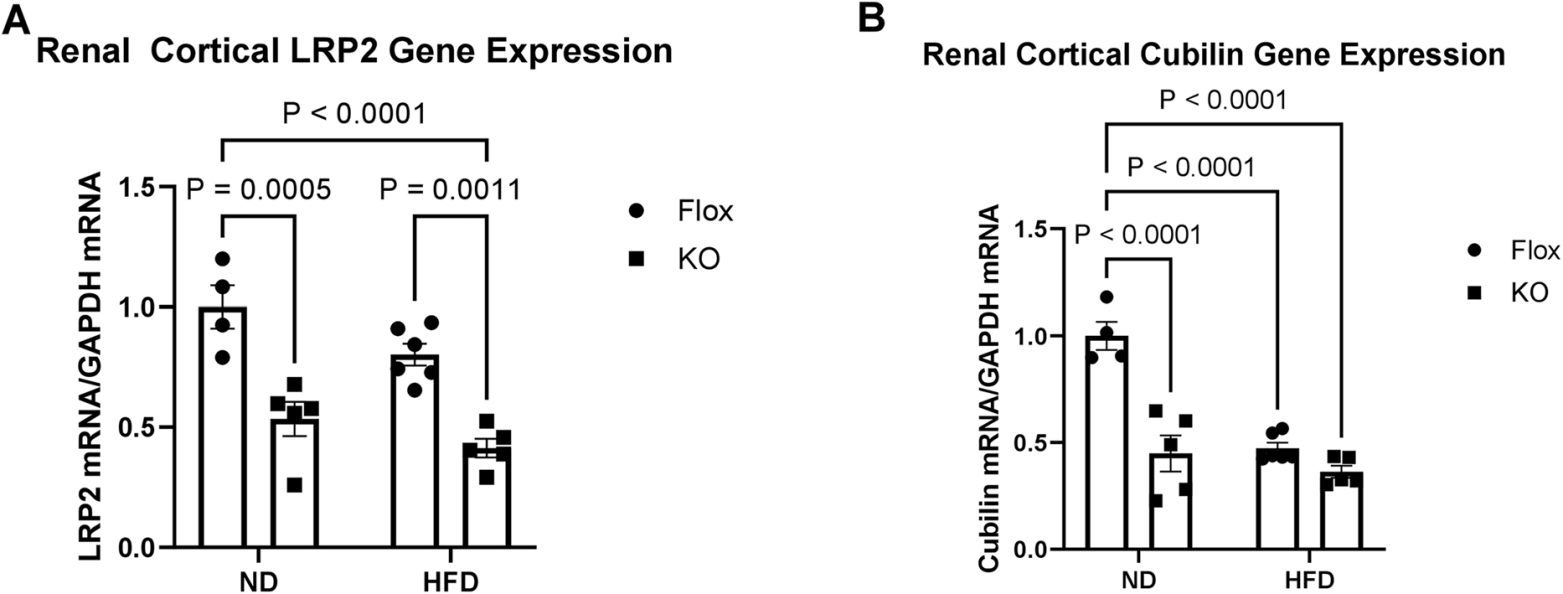
Renal cortical LRP2 and cubilin mRNA expression. In mice with and without ATP6AP2 knockout after 6 months on ND vs HFD. (**A**) RT-PCR for LRP2 mRNA normalized to GAPDH mRNA in renal cortical tissue. ND (N = 4), NDKO (N = 5), HFD (N = 6), HFDKO (N = 5). (**B**) RT-PCR for Cubilin mRNA normalized to GAPDH mRNA in renal cortical tissue. ND (N = 4), NDKO (N = 5), HFD (N = 6), HFDKO (N = 5). Data presented as mean ± SEM and normalized to ND.

**Figure 3:**
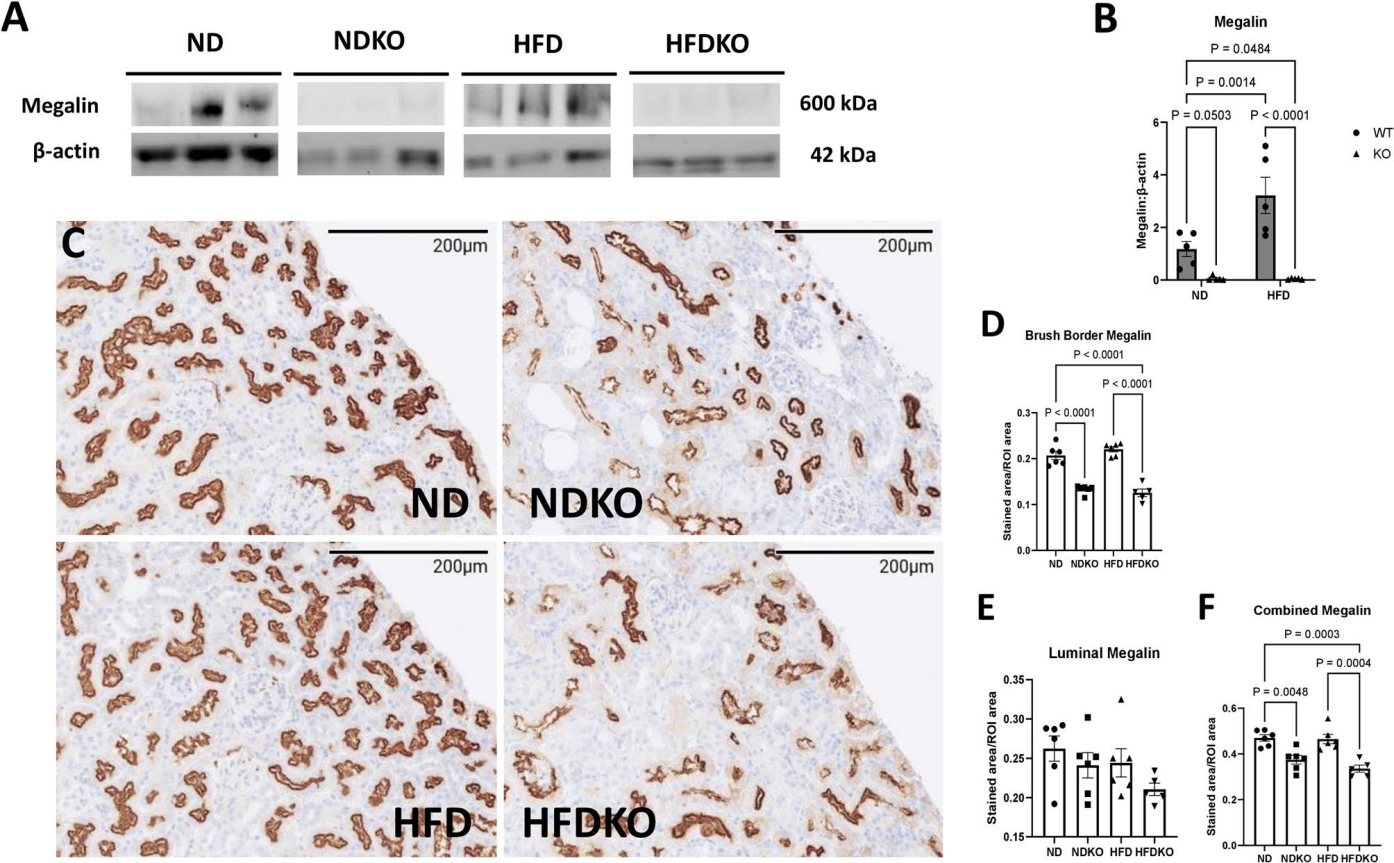
Renal cortical megalin expression. (**A**) Representative Western Blot images of bands for megalin and β-actin. (**B**) Quantitation of megalin signal normalized to β-actin. ND (N = 5), NDKO (N = 5), HFD (N = 5), HFDKO (N = 5). (**C**) Representative light microscopy images of kidney immunohistochemistry for megalin from each treatment group. (**D**) Quantitation of proximal tubule brush border (dark brown) staining expressed as average stained area per region of interest (ROI) area based on analysis of 6 ROIs per section. (**E**) Quantitation of proximal tubule luminal (light brown) staining expressed as average stained area per region of interest (ROI) area based on analysis of 6 ROIs per section. (**F**) Quantitation of combined brush border and luminal staining expressed as average stained area per region of interest (ROI) area based on analysis of 6 ROIs per section. Number of mice per treatment group ND (N = 6), NDKO (N = 6), HFD (N = 6), HFDKO (N = 5). Data presented as mean ± SEM.

Renal proximal tubule cells import albumin and NEFA to a large extent through receptor-mediated endocytosis by the megalin–cubilin complex^17,18^. We therefore sought to determine whether megalin expression was decreased in mice with nephron specific ATP6AP2 knockout. RT-PCR for LRP2, the gene for megalin, demonstrated that renal cortical LRP2 expression was reduced by 47% in NDKO mice compared to ND controls (p < 0.001) and 49% in HFDKO mice compared to HFD controls (p < 0.01) (Fig. 2A). There was no significant difference in LRP2 expression between ND and HFD fed controls. Cubilin gene expression was significantly decreased in NDKO mice compared to ND mice (56%, p < 0.001) but there was no significant difference in cubilin expression between the HFD and HFDKO groups though both were similarly decreased compared to ND controls (Fig. 2B). Quantification of renal cortical megalin protein expression by western blot is shown in Fig. 3A,B. While HFD increased renal cortical megalin expression by 2.7-fold, ATP6AP2 KO reduced cortical megalin expression by 91% in NDKO mice compared to ND and 96% in HFDKO compared to HFD (p < 0.001). Further quantification of renal cortical megalin was performed by immunohistochemistry as shown in Fig. 3C–F. Megalin staining was found to include both dark brown staining which corresponded to the location of the proximal tubule brush border and light brown staining which appeared to be luminal (Fig. 3C). Brush border megalin was not changed by HFD compared to ND controls but was reduced by 36% in NDKO mice compared to ND controls (p < 0.001) and 44% in HFDKO mice as compared to HFD controls (p < 0.001) (Fig. 3D). There were no significant differences in luminal megalin staining between groups (Fig. 3E) whereas combined staining showed a 21% decrease in NDKO compared to ND (p < 0.01), and 28% decrease in HFDKO from HFD (p < 0.01) (Fig. 3F).

### Nephron specific ATP6AP2 increases renal cortical mTOR signaling while inhibiting autophagy

**Figure 4:**
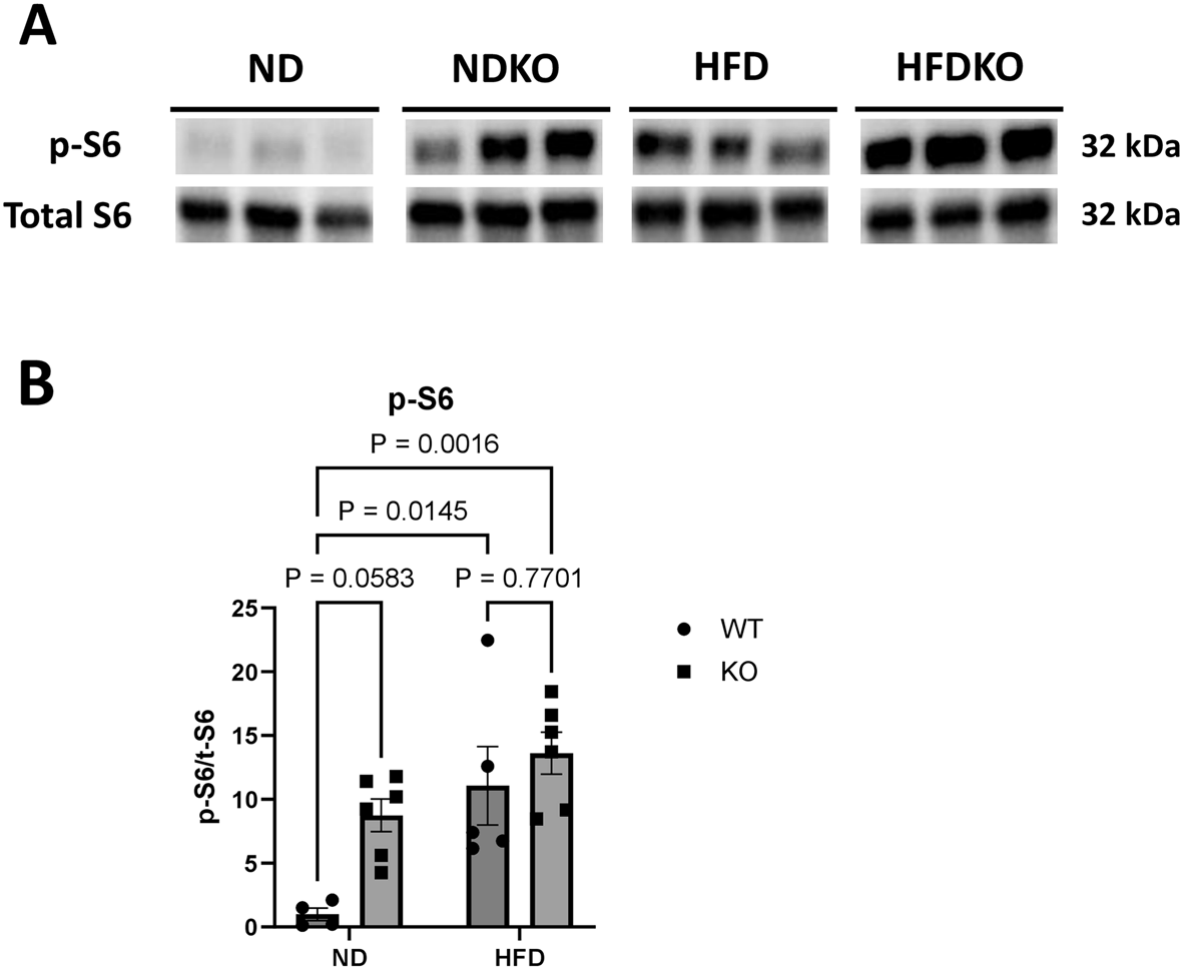
Renal cortical p-S6 expression. (**A**) Representative bands from Western Blot of p-S6 and total-S6 with samples taken from mice with and without ATP6AP2 knockout on ND v. HFD groups after 6 months. (**B**) p-S6 signal normalized to total-S6. ND (N = 5), NDKO (N = 6), HFD (N = 6), HFDKO (N = 6) Data presented as mean ± SEM.

Our group and others have shown that loss of ATP6AP2 can impair autophagy and lysosomal dysfunction has recently been implicated in reducing receptor-mediated endocytosis by the proximal tubule^14,19^. Prior studies have also suggested that ATP6AP2 may regulate megalin expression via mTOR^15^. We therefore evaluated the effect of our nephron specific ATP6AP2 knockout on both mTOR activity and lysosomal function. We measured renal cortical phosphorylation of the ribosomal protein S6, an accepted downstream marker of mTOR activation^20^, by western blot as shown in Fig. 4A. HFD increased levels of p-S6 by over nine-fold (p < 0.05) compared to ND controls. Interestingly, NDKO mice had a nearly ten-fold increase in p-S6 (p = 0.06) compared to ND controls while HFDKO mice demonstrated a non-significant increase of 22% in p-S6 compared to HFD controls (p = 0.77) (Fig. 4B).

**Figure 5:**
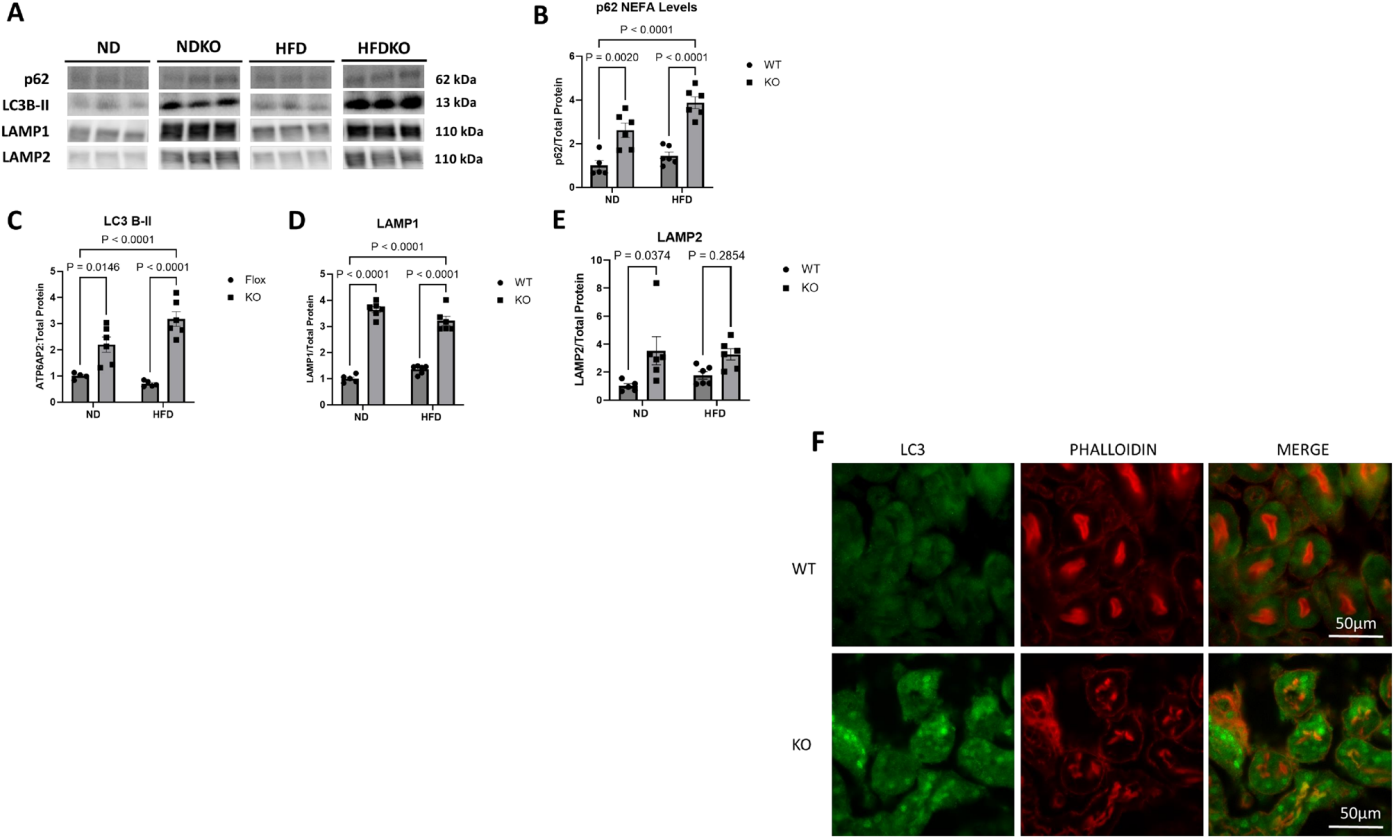
Renal cortical expression of autophagy markers. (**A**) Representative bands from Western Blot of p62, LC3B-II, LAMP1, and LAMP2. (**B**) p62 signal normalized to total protein. (**C**) LC3B-II signal normalized to total protein. (**D**) LAMP1 signal normalized to total protein. (**E**) LAMP2 signal normalized to total protein. ND (N = 5), NDKO (N = 6), HFD (N = 6), HFDKO (N = 6). Data presented as mean ± SEM. (**F**) Confocal microscopy of immunofluorescence for LC3B in renal cortex of ND and NDKO mice. Green color represents positive staining for LC3B and red staining represents positive staining for proximal tubule marker phalloidin. See originally produced, full-length blots in Supplementary Figures.

Prior studies have shown an increase in LC3B-II and Lamp1, corresponding to accumulation of autophagosomes and lysosomes in nephron specific ATP6AP2 knockout mice^14^. We therefore conducted western blot for expression of LC3B-II and LAMP1 as well as additional autophagy markers p62 and LAMP2 (Fig. 5A–E). While there was no significant difference in expression of any of these markers between ND and HFD controls, there were nonsignificant trends toward increased p62, LAMP1 and LAMP 2 in HFD compared to ND. In contrast, ATP6AP2 knockout significantly increased all four markers under ND conditions. Under HFD conditions, ATP6AP2 knockout significantly increased p62, LC3B-II, and LAMP1 with a nonsignificant increase observed for LAMP2. Immunofluorescent staining for LC3B (Fig. 5F) further demonstrated increased accumulation of LC3B in the proximal tubule. These results strongly indicate autophagic dysfunction with ATP6AP2 knockout in the proximal tubule.

## Discussion

These results demonstrate that ATP6AP2 knockout in the renal nephron results in decreased proximal tubule megalin expression and increased urinary NEFA excretion in the setting of impaired autophagy. To our knowledge, this is the first time that renal ATP6AP2 has been implicated in lipid handling by the kidney. We recently reported that nephron specific ATP6AP2 knockout causes impaired weight gain on HFD accompanied by increased urinary excretion of other metabolites including glucose and albumin^10^. In the same study, we also found that the knockout mice did not have lower caloric intake or higher basal metabolic rate, indicating that urinary loss of calories was the most likely explanation for their lower weight. Lipids are a more concentrated source of energy than carbohydrate or protein and so urinary excretion of fatty acids could be an additional contributor to the inability of these mice to gain weight despite being on HFD.

Our results are notable first for the fact that a comparable decrease in megalin occurred under both ND and HFD conditions and corresponded to a similar degree of albuminuria. Renal inflammation has been shown to decrease renal megalin levels under conditions of high salt feeding^21^. Though different from high salt conditions, HFD induced obesity is also a proinflammatory state. Nonetheless, in our study HFD alone had no significant effect on either megalin gene expression or megalin protein level when measured by immunohistochemistry. The effects on megalin seen in high salt diet, however, were largely related to internalization and degradation of megalin rather than changes in gene expression. Our knockout mice, however, had drastically decreased LRP2 gene expression. This aligns with studies in drosophila showing that ATP6AP2 knockdown reduces LRP2 gene expression^15^. Additionally, the increase in albumin:creatinine ratio with HFD alone was modest and not statistically significant compared to ND controls while the albuminuria induced by ATP6AP2 knockout was not greater under HFD conditions than ND. This would seem to indicate no additive effect of HFD and ATP6AP2 knockout in albuminuria. This was somewhat surprising given that obesity has been reported to result in glomerular hyperfiltration of albumin and fatty acid^4,8^. However, it is possible that the significance of the increase in albuminuria under HFD conditions was simply much smaller in magnitude compared to the effect of the knockout.

It is well established that fatty acids are detectable in the urine of even healthy individuals and glomerular filtration of albumin bound fatty acids is thought to be the primary mechanism of excretion^22^. Proximal tubule epithelial cells absorb filtered albumin along with bound fatty acids via receptor-mediated endocytosis in which the megalin/cubilin complex binds and internalizes albumin into cellular endosomes^17,18^. In line with this, megalin knockout in mice results in increased albuminuria^23^. One of the cleavage products of the ATP6AP2 gene is an accessory subunit of the cellular vacuolar H^+^-ATPase^13,24^. Loss of ATP6AP2 in drosophila decreases H^+^-ATPase activity, resulting in in impaired receptor-mediated endocytosis via reduced megalin expression^15^. It is therefore logical that ATP6AP2 knockout in the renal tubular epithelium would result in decreased megalin expression and albuminuria leading to increased urinary NEFA excretion.

Prior investigation by Figueiredo et al. using a similar nephron specific ATP6AP2 knockout model also found reduced receptor-mediated endocytosis and albuminuria^14^. However, their study did not show any differences in renal megalin expression and instead attributed these changes entirely to a loss of lysosomal function. The current study also found elevation in LC3B-II, p62, LAMP1, and LAMP2 with ATP6AP2 knockout, indicating impaired autophagy and this is consistent with previous reports by our laboratory that ATP6AP2 knockdown impairs autophagy in podocytes^19^. Evidence exists that impaired autophagy can decrease megalin through reduced recycling of the receptor to the cell surface^25^. While this mechanism may help to explain the lower megalin levels in our knockout, our data further demonstrate decreased LRP2 expression which would indicate that megalin gene expression is affected as well. At the same time, the results of this study also suggest an increase in mTOR signaling with ATP6AP2 knockout. This was surprising as others had previously proposed that impairment of the vaculoar V-ATPase decreases megalin expression via reduction in mTOR^15^. Notably, Figueiredo et al. did not identify any changes in mTOR activation with ATP6AP2 knockout in the renal nephron^14^ and the increase in mTOR activity observed in our study is actually consistent with our other findings as mTOR signaling inhibits autophagy^26^. Nonetheless, these results indicate that ATP6AP2 may regulate LRP2 activity via an alternative mechanism.

It should also be noted that not all investigations into ATP6AP2 in the proximal tubule have reported impairment in proximal tubule autophagy. One study of a similar inducible nephron specific ATP6AP2 knockout mouse reported impaired autophagy in the medulla but not cortex^27^. While the reason for this discrepancy is not entirely clear, the model used in that study had significant differences as knockout was induced in utero. This could have affected renal development as ATP6AP2 is necessary in normal kidney embryology^28,29^. Taken together, the current findings are nonetheless consistent with those of Figueiredo et al. and our own findings in other kidney cell types.

Our findings also have implications for the overall understanding of obesity related kidney injury. Greater weight is an independent risk factor for progression of chronic kidney disease in human subjects^3,30,31^. In addition to hypertension and hyperglycemia, dyslipidemia and lipotoxicity are thought to play an important role in the pathophysiology of obesity related kidney disease^32^. Studies of obesity in both mice and humans have demonstrated renal ectopic lipid accumulation, including renal proximal tubule epithelial cells, associated with increased kidney injury and renal fibrosis^4,33^. In the same way, measures which reduce proximal tubule lipid accumulation may prevent renal proximal tubule injury^34–36^. Renal megalin knockout has been shown to decrease proximal tubule lipid accumulation and renal fibrosis though that study did not assess whether urinary fatty acid excretion was also increased^23^. More globally, ATP6AP2 knockout has been shown to reduce lipid accumulation in other organ systems, including liver and adipose, and has been further implicated in altering intracellular lipid metabolism including reduction in lipid synthesis^37,38^. However, the role of ATP6AP2 in renal lipid metabolism remains unclear. Additionally, there is evidence that different lipid species can have a toxic or protective effect in the renal proximal tubule but it is not known whether ATP6AP2 has a role in determining renal lipid composition as well^39^. It would therefore be important in future investigation to assess the effect of ATP6AP2 knockout on proximal tubule lipid accumulation and renal lipotoxicity.

There are also several notable limitations to the current study. For one, our methods do not directly assess the uptake of NEFA by receptor mediated endocytosis nor the relative contribution of glomerular filtration versus impaired proximal tubule reabsorption in the observed albuminuria and fatty acid excretion. We have previously assessed this knockout model for evidence of glomerular injury using electron microscopy and did not find evidence of significant podocyte effacement^10^. As mentioned, others have produced evidence to support the idea that loss of ATP6AP2 reduces receptor-mediated endocytosis in the proximal tubule, indicating that this aspect of proximal tubule dysfunction is more likely responsible for the increase in NEFA excretion^14^.

Secondly, our model utilizes ATP6AP2 knockout throughout the renal nephron whereas the focus of the current study primarily implicates changes to the proximal tubule. We and others have previously reported that ATP6AP2 knockout has significant distal tubule effects on sodium and water handling^16,40^. While it is established that changes in one tubule segment can effect changes in other segments, it is unlikely that this played a role in the current study as reabsorption of albumin and fatty acids occurs exclusively in the proximal tubule^41,42^. Nonetheless, future investigation using a proximal tubule specific knockout model would be helpful in validating these findings. Finally, receptor-mediated endocytosis by the megalin/cubilin complex is not the only mechanism by which proximal tubule cells take up lipid. Additional transport molecules including fatty acid binding protein, fatty acid transport proteins, and CD36 assist in importing fatty acids and increased CD36 is associated with lipotoxity^43–45^. While the current study did not assess the role of these additional transporters, further study of the role of ATP6AP2 in renal lipid transport should determine whether these pathways are involved.

In conclusion, nephron specific knockout of ATP6AP2 results in increased urinary excretion of fatty acid in association with decreased proximal tubule megalin expression. This raises important future questions, not only regarding the function of ATP6AP2 in renal lipid transport, but also its role in proximal tubule lipid accumulation in the setting of obesity related kidney injury.

## Materials and methods

### Animal care and use

All study protocols were approved by the University of Virginia Animal Care and Use Committee and were conducted in accordance with the Public Health Service Policy on Humane Care and Use of Laboratory Animals and guidelines as described by the NIH Guide for the Care and Use of Laboratory Animals as well as in accordance with ARRIVE guidelines. All studies utilized inducible nephron specific ATP6AP2 knockout mice and noninduced littermate controls on a C57BL/6J background. The inducible ATP6AP2 knockout mouse was generously provided by Drs. Donald Kohan and Nirupama Ramkumar at the University of Utah Health Sciences Center in Salt Lake City, UT. As previously described, these mice are homozygous for a floxed ATP6AP2 gene and are homozygous for Pax8-rTA and LC1 Cre^16^.

### Genotyping

All mice were genotyped at 3 weeks of age using genomic DNA from tail snips. Tail samples were digested in an alkaline digestion buffer and then underwent PCR using the following primers: ATP6AP2 forward: 5′-GGGGGGTAAATTGTTGATGAGTCTTGGAGCATAGC-3′; reverse 5′-GAAGCCCATGGACAGTGCAGCTACGTCTGGGATTCGA-3′; PAX-8-rtTA forward: 5′-CCATGTCTAGACTGGACA AGA-3′; reverse: 5′-CTCCAGGCC ACATATGAT TAG-3′; LC-1 forward: 5′-TCGCTGCATTACCGGTCGATGC-3′; reverse 5′-CCATGAGTGAACGAACCTGGTCG-3′. PCR products were separated by electrophoresis on a 1% agarose gel and imaged under UV light.

### Mouse diet treatment, metabolic assessment, and organ collection

All mice were housed on 12-h light–dark cycle with ad libitum access to water. ATP6AP2 knockout was induced at 8 weeks of age using oral administration of 2 mg/mL doxycycline (Sigma, St. Louis, MO) in 2% sucrose water for 12 days. Control mice received 2% sucrose water only over the same period. At 10 weeks of age, mice were randomly assigned to either standard chow (12% fat) (Teklad, Indianapolis, IN) or HFD (45% fat from lard) (Research Diets, D12451, New Brunswick, NJ) for 6 months. This generated 4 treatment groups including normal diet (ND) and HFD controls as well as normal diet knockout (NDKO) and high fat diet knockout (HFDKO). After 5 months of diet, all mice underwent 48-h metabolic cage assessmentwith the first 24 h used to condition the mice to the cages followed by data collection over the subsequent 24 h. In this procedure, mice were weighed and then placed in individual metabolic cages. Food and water in the cage were weighed at the beginning and end of the 24-h measurement period to determine 24- hour food and water consumption. 24-h caloric consumption was calculated from 24-h food consumption by multiplying the measured weight of food consumed by 3.1 kcal/g and 4.73 kcal/g for normal diet and high fat diet respectively. Urine was collected during the 24-h measurement period to determine urine volume and urine aliquots were then stored at – 80 °C until analysis. Blood samples were obtained in the morning under random fed conditions through retroorbital blood draw. Blood was allowed to coagulate at room temperature for 30 min, centrifuged at 4 °C for 10 min at 2000 RCF, followed by extraction of the serum supernatant. Serum was stored at – 80 °C prior to analysis. Serum triglyceride levels were measured using Wako triglyceride assay (Fujifilm).

For insulin tolerance testing, mice were fasted for 4 h prior to the start of testing. Throughout the test, blood glucose was measured with blood obtained by tail snip using a Contour Next glucometer (Bayer). Blood glucose was measured at the start of the test and then mice were injected with 1 mUnit/g body weight regular insulin via peritoneal injection. Blood glucose was then measured at 15, 30, 45, and 60 min post-injection.

At the end of 6 months, mice were anesthetized with ketamine and xylazine and euthanized. Kidneys were immediately removed, decapsulated and sectioned. Samples for protein and RNA analysis were separated into cortical and medullary components followed by snap freezing at – 80 °C. Samples for immunohistochemistry were processed as described below.

### Transdermal GFR measurement and plasma creatinine measurement

For transdermal measurement of GFR, mice were first shaved 48 h prior to testing.

On the day of testing, a transdermal fluorescence detector (Medi-Beacon) was placed on the mouse’s flank. The mouse was then administered 0.15 mg/g body weight FITC-Sinistrin (Medi-Beacon) via tail vein injection and transdermal fluorescence was recorded for 1 h post-injection in the unanesthetized mouse. Data from the device was analyzed using medibeacon studio software to calculate GFR as previously described^46^. For Plasma creatinine measurement, whole blood was obtained via retroorbital bleeding and placed in a tube with EDTA. Samples were centrifuged for 10 min at 2000*g*. Plasma creatinine was then quantitated in supernatant using Diazyme Creatinine Liquid Reagent Assay (Diazyme).

### RT-PCR

Renal cortical samples were lysed in Trizol (Invitrogen, Carlsbad, CA) and RNA was isolated from kidney tissue samples using a Direct-zol RNA miniprep kit (Zymo, Irving, CA). Reverse transcription was carried out using an iScript cDNA synthesis kit (Bio-Rad, Hercules, CA). PCR was then performed using SSO Advanced Universal SYBR Green Supermix (Bio-Rad) on a Bio-Rad CFX Connect Real-Time System. Reactions were performed in duplicate and normalized to GAPDH mRNA. Primer sequences were as follows: ATP6AP2 forward 5′-TCTCCGAACTGCAAGTGCTA-3′; reverse 5′-CTGCAAACTTTTGGAGAGCA-3′; LRP2 forward 5′-TGTGAACACCAGCCTGGTTT-3′; reverse 5′-TGGAGCCCGTGAGAGTACTG-3′; Cublin forward 5′-TGGGATCTCCTGGAAATGAG-3′; reverse 5′-ACCGCTTGGGTAGACATTTG-3′; GAPDH forward 5′-CCAGGTTGTCTCCTGCGACT-3′; reverse 5′-ATACCAGGAAATGAGCTTGACAAAGT-3′.

### Western blot

Renal cortical tissue samples were homogenized in T-PER tissue protein extraction reagent (Thermofisher, Waltham, MA) with addition of HALT protease inhibitor (Thermofisher). Homogenate was centrifuged for 10 min at 900*g* at 4C and BCA assay was performed on supernatant to determine protein concentration. For standard western blot of proteins other than megalin, samples were prepared in Laemmli buffer and 30 µg of protein were utilized for all assays. Samples were separated by electrophoresis on 10% Criterion TGX Stain-Free gel (Bio-Rad) transferred to a low-flouresence polyvinyldene difluoride membrane (Bio-Rad) by Trans-blot Turbo Transfer System (Bio-Rad). Membranes were blocked with 5% milk in 1X TBST with 0.1% Tween for 1 h at room temperature then incubated with primary antibody in 5% BSA in 1X TBST overnight at 4 °C. Blots for phosphorylated proteins were then incubated with secondary antibody in 5% BSA in 1X TBST for 1 h at room temperature while blots for nonphosphorylated proteins were incubated with secondary antibody in 5% milk in 1X TBST in the same manner. Immunoreactive bands and total protein was detected using a Chemidoc MP imager (Bio-Rad). Densitometric analysis was performed using Image Lab Software (Bio-Rad). Phosphorylated protein quantitation was normalized to the corresponding total protein while nonphosphorylated protein was normalized to total protein. Antibodies for western blot included anti-p-S6 (1:1000, 2211, Cell Signaling, RRID:AB_331679), anti-s6 (1:1000, 2217, Cell Signaling, RRID:AB_331355), anti-LC3BA/B (1:1000, 4108, Cell Signaling, RRID:AB_2137703), anti-LAMP1 (1:500, ab25245, Abcam, RRID:AB_449893), anti-LAMP2 (1:200, ab13524, Abcam, RRID:AB_2134736), anti-SQSTM1/p62 (1:1000, 5114, Cell Signaling, RRID:AB_10624872), anti-rabbit-HRP (1:1000, ab97051, Abcam, RRID:AB_10679369), anti-rat-AlexaFluor 647 (1:1000, A21247, Thermo Fisher Scientific, RRID:AB_141778). For western blot detection of the high molecular weight protein megalin, samples were prepared in XT sample buffer with XT reducing agent (Bio-Rad). Samples were separated by electrophoresis on a 3–8% Criterion Tris–Acetate gel (Bio-Rad) and transferred to a low-flouresence polyvinyldene difluoride membrane (Bio-Rad) via overnight wet transfer at 4 degrees Celsius. Blocking and antibody incubation procedure was the same as for standard western with anti-megalin primary antibody (1:500,SC-515772, Santa Cruz Biotechnology, RRID:AB_2783023) and anti-mouse-HRP secondary (1:1000, 7076, Cell Signaling, RRID:AB_330924). For megalin, protein quantitation was normalized to beta-actin detected using anti-actin hFAB rhodamine antibody (1:5000, 12004163 Bio-Rad, RRID:AB_2861334).

### Immunohistochemistry and immunofluorescence

For immunohistochemistry, kidney samples were fixed for 24 h in 10% buffered formalin (Thermo Scientific, Waltham, MA) and then transferred to 70% ethanol. Samples then underwent paraffin embedding and sectioning at the University of Virginia Research Histology Core. Immunohistochemistry staining was performed on a robotic platform (Ventana discover Ultra Staining Module, Ventana Co., Tucson, AZ, USA) at the University of Virginia biorepository and tissue facility. Tissue sections of 4 μm thickness were deparaffinized using EZ Prep solution (Ventana). A heat-induced antigen retrieval protocol set for 64 min was carried out using Cell Conditioner 1 (Ventana). Endogenous peroxidases were blocked with peroxidase inhibitor (CM1) for 8 min before incubating the section with anti-megalin antibody (1:200, ab76969, abcam, RRID:AB_10673466) for 60 min at room temperature. Antigen–antibody complex was detected using a DISCOVERY OmniMap anti-rabbit multimer RUO detection system and DISCOVERY ChromoMap DAB Kit (Ventana Co.). All the slides were then counterstained with hematoxylin, dehydrated, cleared, and mounted for assessment. Visiopharm Oncotopix software was used to quantify positive megalin staining as follows. All stained tissue sections were captured using NanoZoomer S360 high-resolution scanner (Hamamatsu, Japan) at 40 × scan magnification. The whole slide images were transferred to an analysis platform that hosted the Oncotopix software (Visiopharm A/S, Hoersholm, Denmark). To extract quantitative information from images, thresholding criteria was used for accurate megalin detection and segmentation. Appropriate features and smoothening filters were applied as preprocessing steps to improve segmentation quality. The algorithm was then trained across several images from the project to account for sample variability, reduce artifacts and detect all positive staining. In our previous work with this model, knockout mice were noted to have areas of abnormal hypercellularity within the cortex^10^. For this analysis, hypercellular areas were highlighted by the principle investigator and excluded from the ROIs or analysis of megalin expression. A total of six ROIs were used for the analysis as technical replicates. The ROIs of equal area were drawn equidistant from each other to avoid any bias. The first ROI was arbitrarily defined by the proximal (top) end of the mouse kidney and the rest were drawn/copied accordingly.

For immunofluorescence, kidney samples were fixed for 2 h in 4% paraformaldehyde (Alfa Aesar, Haverhill, MA). Samples were washed in phosphate buffered saline, treated with 100 mM tris–HCl for 1 h, followed by overnight treatment with 30% sucrose. Tissue was then frozen in OCT and cut in 8-micron sections on slides. Sections were permeabilized in 0.3% Triton-X in TBS, blocked with Odyssey Blocking Buffer (Li-Cor Biosciences, Lincoln NE) for one hour at room temperature, and probed with anti-LC3BA/B antibody (1:100, 4108, Cell Signaling, RRID:AB_2137703) overnight at 4 degrees Celsius. Sections were then exposed to Alexa-647 donkey anti-rabbit antibody (1:500, A31573, Thermo Fisher Scientific, RRID:AB_2536183) and Alexa-750 anti-Phalloidin antibody (1:200, A30105, Thermo Fisher Scientific) for one hour at room temperature, washed, and mounted in Fluoromount (Electron Microscopy Sciences) Images were taken using an Olympus IX81 spinning disk confocal microscope using a 60 × plan apochromat water immersion objective with a numeric aperture of 1.2. Background staining was gated using sections exposed to secondary antibody only.

### Urine albumin-creatinine and urine NEFA assessment

Urine albumin to creatinine ratio was calculated using the urine albumin concentration measured with the Albuwell M Mouse Albumin Elisa (Exocell, Newton Square, PA) and the urine creatinine concentration measured by a urine creatinine colorimetric kit (Cayman Chemical Company, Ann Arbor, MI). For Urine NEFA measurements, urine samples underwent a Folch organic extraction as follows. Four hundred microliters of urine were mixed with 1 mL of methanol and 0.5 ml of chloroform. Following one hour of incubation on ice, 0.5 mL of 0.2 molar NaCL was added and samples were centrifuged at 1000*g* for 1 min. The organic layer was then extracted, dried under nitrogen gas, followed by resuspension in 0.05% fatty acid free BSA solution. NEFA concentration was then measured in each suspension using a fluorometric free fatty acid quantitation kit from sigma (MAK044).

### Statistical analysis

All comparisons between multiple treatment groups were carried out using 2-way ANOVA with Tukey test for comparisons. Statistical analyses were carried out using GraphPad Prism. Data are expressed as mean ± standard error of the mean and *P* values < 0.05 are considered statistically significant.

### Supplementary Information

Supplementary Figures.

## Data Availability

The datasets generated during and/or analysed during the current study are available from the corresponding author on reasonable request.
