# Supplementary material for: Nephron specific ATP6AP2 knockout increases urinary excretion of fatty acids and decreases renal cortical megalin expression
Source: Sci Rep. 2024 Aug 12;14:18724. doi: 10.1038/s41598-024-69749-x (PMC11319469; doi:10.1038/s41598-024-69749-x)
Supplement: Supplementary file 1 — Supplementary Figures. [file 41598_2024_69749_MOESM1_ESM.docx]

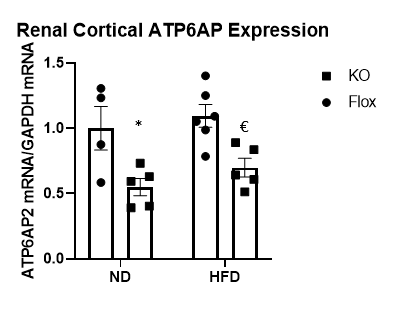


**Supplementary Figure 1. Renal Cortical ATP6AP2 Expression** RT-PCR for ATP6AP2 mRNA normalized to GAPDH mRNA in renal cortical tissue after 6 months on ND vs HFD with and without ATP6AP2 knockout. ND (N=4), NDKO (N=5), HFD (N=6), HFDKO (N=5). Data presented as mean ± SEM and normalized to ND. *p<0.05 compared to ND, €p<0.05 compared to HFD.


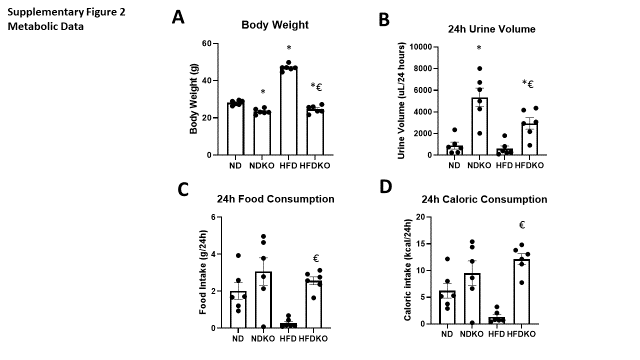


**Supplementary Figure 2. Mouse body weight and Metabolic Data.** (A) Mouse body weights after 6 months on ND vs HFD with and without ATP6AP2 knockout, (B-D) Urine volumes, food consumption, and caloric consumption obtained by 24h metabolic cage after 6 months on ND vs HFD in mice with and without ATP6AP2 knockout. ND (N=6), NDKO (N=6), HFD (N=6), HFDKO (N=6). Data presented as mean ± SEM. *p<0.05 compared to ND, €p<0.05 compared to HFD.


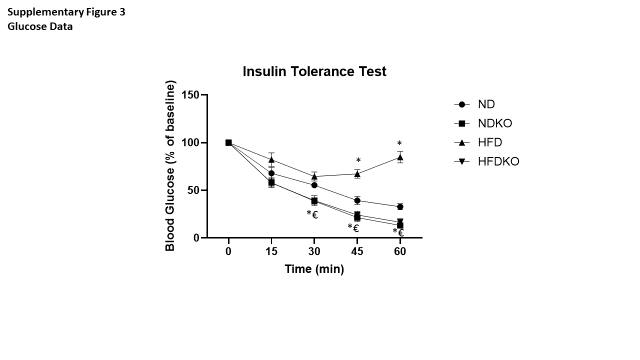


**Supplementary Figure 3. Insulin Tolerance Testing.** Insulin tolerance test performed by injecting mice following 4 hour fast with 1miliunit/g body weight insulin at time 0 with blood glucose monitored at 15 minute intervals using contour next glucometer wild type in ND vs. HFD fed mice with and without nephron specific ATP6AP2 knockout after 6 months on diet. Data expressed as % of baseline blood glucose. ND (N=5), NDKO (N=7), HFD (N=6), HFDKO (N=6). *p<0.05 compared to ND, €p<0.05 compared to HFD.

**
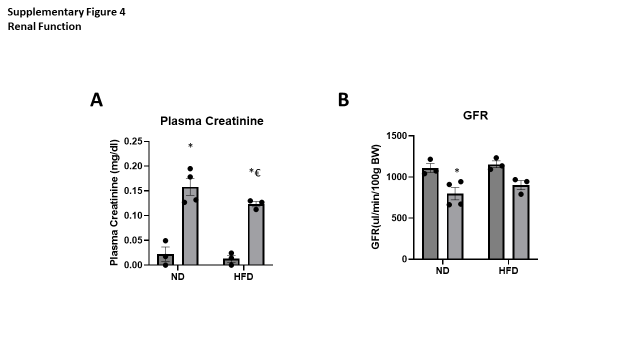
Supplementary Figure 4. Renal Function** . Plasma creatinine determined using plasma creatinine kit from Diazyme, performed on plasma from ND vs. HFD fed mice with and without nephron specific ATP6AP2 knockout after 6 months on diet. Glomerular filtration rate determined using transdermal detection of FITC-sinistrin contrast clearance over 1 hour after tail vein injection. Procedure was performed using transdermal detectors from medibeacon with glomerular filtration calculation using MB Studio software from medibeacon. ND (N=3), NDKO (N=4), HFD (N=3), HFDKO (N=3). *p<0.05 compared to ND, €p<0.05 compared to HFD.

**
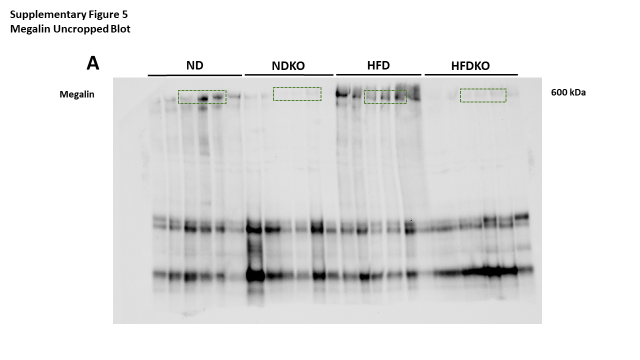
** **
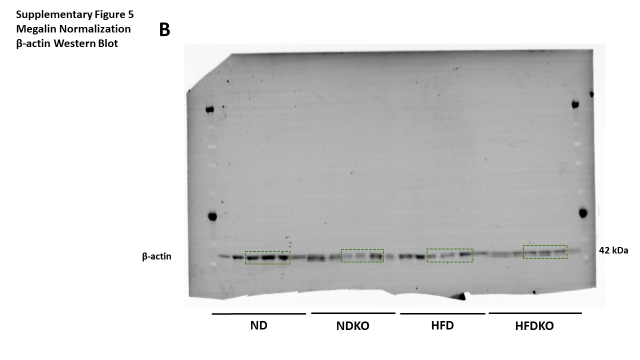
**

**Supplementary Figure 5. Megalin Western Blot.** (A-B) Full-length western blot image of megalin-assayed mouse renal cortex samples. The samples includes ND v. HFD mice with and without ATP6AP2 knockout. Bands were observed in 600 kDa (supplementary fig. 5A) with normalization to β-actin bands shown in 42 kDa (supplementary fig. 5B). Highlighted bands were used as representative bands in manuscript figure 3E.

**
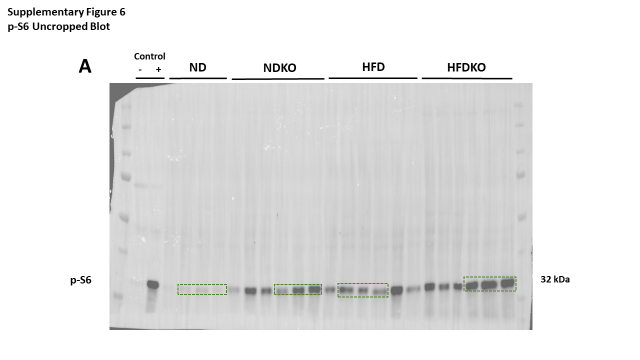

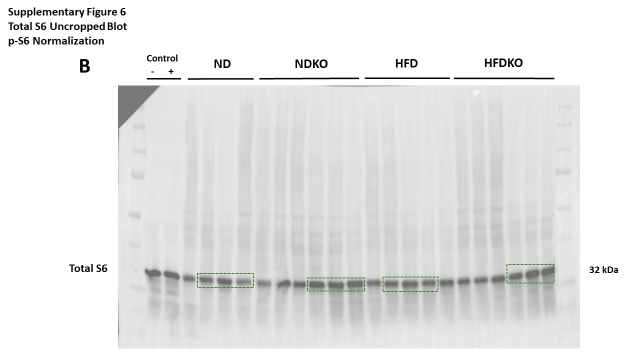
**

**Supplementary Figure 6. p-S6 Western Blots.** (A-B) Full-length western blot image of p-S6 and total S6 with cell lysate controls and mouse renal cortex samples. Controls used for this blot were starved HeLa cell lysate as negative controls and insulin-treated HeLa lysate as positive controls. Mouse renal cortex samples include ND v. HFD mice with and without ATP6AP2 knockout. Bands are ~32 kDa for both total and p-S6. P-S6 (supplementary fig. 1A) bands were normalized to total-S6 (supplementary fig. 1B) bands. Highlighted bands were used as representative bands in manuscript figure 6A.


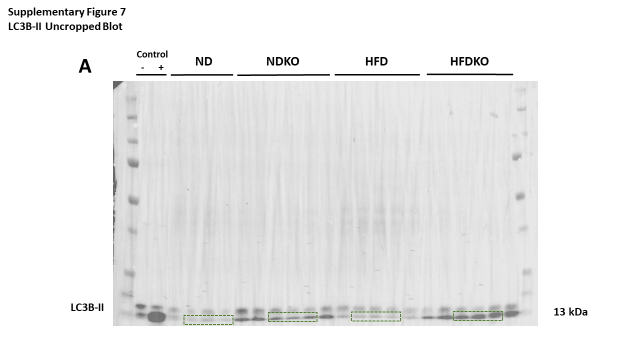

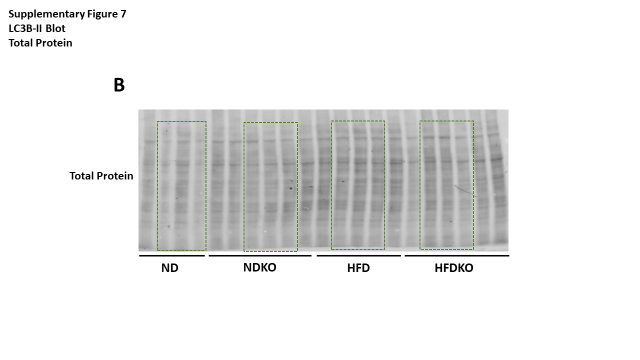


**Supplementary Figure 7. LC3B-II Western Blots.** (A-B) Full-length western blot image of LC3B-II and total protein with cell lysate controls and mouse renal cortex samples. Controls used for this blot were commercially acquired HeLa cells treated with 50 µM chloroquine as positive control and untreated HeLa cells as negative control (11972, Cell Signaling). Mouse renal cortex samples include ND v. HFD mice with and without ATP6AP2 knockout. . Highlighted bands and lanes were used as representative bands and lanes in manuscript figure 6C.


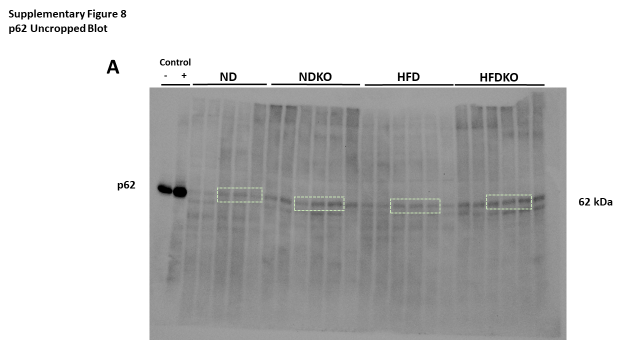

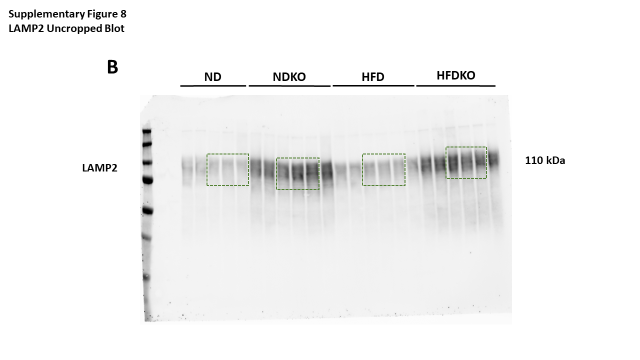

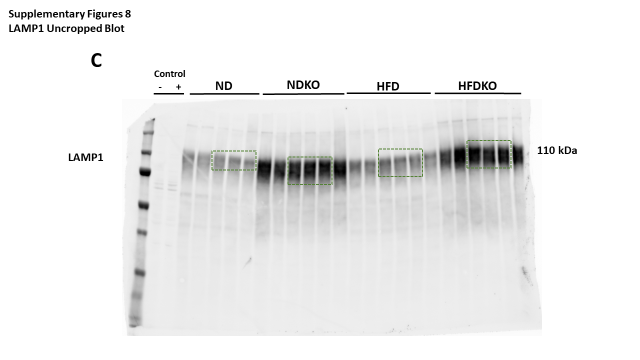

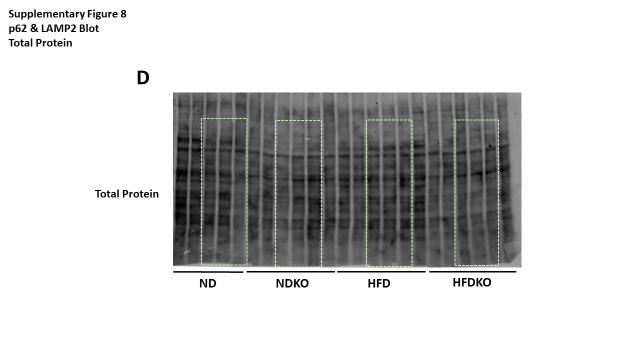

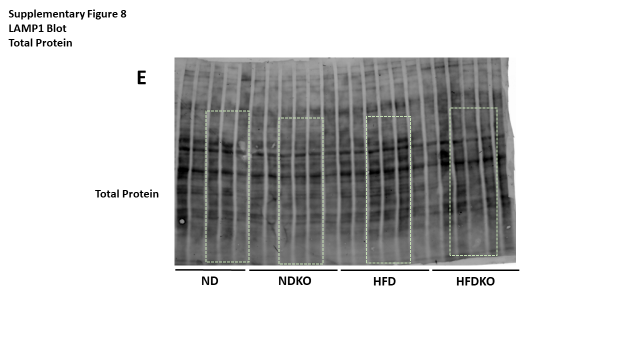


**Supplementary Figure 8. Western blots for autophagy markers.** (A-C) Full-length images of western blots for autophagy markers: p62 and LAMP1-2. (D-E) Total protein for each blot was used to normalize bands for analysis. Controls used for immunoassays were HeLa cells treated with 50 µM chloroquine as positive control and untreated HeLa cells as negative control (11972, Cell Signaling). Murine renal cortical protein samples were used for these assays.
